# Postnatal resveratrol supplementation improves cardiovascular function in male and female intrauterine growth restricted offspring

**DOI:** 10.14814/phy2.13109

**Published:** 2017-01-20

**Authors:** Amin Shah, Anita Quon, Jude S. Morton, Sandra T. Davidge

**Affiliations:** ^1^ Department of Obstetrics and GynecologyUniversity of AlbertaEdmontonAlbertaCanada; ^2^ Women and Children's Health Research InstituteUniversity of AlbertaEdmontonAlbertaCanada; ^3^ Department of PhysiologyUniversity of AlbertaEdmontonAlbertaCanada

**Keywords:** Cardiac function, IUGR, resveratrol

## Abstract

Intrauterine growth restriction (IUGR) may predispose offspring to an increased susceptibility of developing cardiovascular disease (CVD) in adult life. The window of opportunity to treat later life CVD programmed in fetal life is critical. The aim of this study was to identify the effect of resveratrol treatment of IUGR offspring at a time of known CV dysfunction. Sprague–Dawley male and female rat offspring who experienced normoxia (21% O_2_; control) or hypoxia (11% O_2_; IUGR) in utero were fed a high‐fat (HF) diet (3–21 weeks of age) or a HF diet (3–21 weeks of age) supplemented with resveratrol from 13 to 21 weeks of age. At 21 weeks of age, echocardiographic data showed that male IUGR offspring had mild in vivo diastolic dysfunction, whereas female IUGR offspring had early signs of cardiac diastolic dysfunction that was not altered by resveratrol treatment. Notably, male and female IUGR offspring demonstrated equal susceptibility to ex vivo cardiac dysfunction recovery after ischemia/reperfusion (I/R) injury and this was improved by resveratrol treatment, independent of sex. Resveratrol increased cardiac phospho‐adenosine monophosphate kinase (p‐AMPK) levels in only female IUGR offspring. IUGR or resveratrol did not alter cardiac superoxide levels. However, in male offspring, an overall effect of IUGR in reducing cardiac catalase levels was observed that was not altered by resveratrol. Interestingly, in only female IUGR offspring, resveratrol significantly increased cardiac superoxide dismutase (SOD) 2 levels. In conclusion, resveratrol treatment of adult IUGR offspring, at the time of known CV dysfunction, improved cardiac function recovery in both sexes and the mechanisms involved were partially sex‐specific.

## Introduction

Cardiovascular disease (CVD) is one of the most prevalent diseases and is the leading cause of mortality worldwide (Roth et al. 2015). A link between low birth weight due to pregnancy complications and CVDs in later life has been identified and explored for several decades (reviewed in (Alexander et al. 2015)). Fetal hypoxia, one of the most common outcomes of pregnancy complications, may lead to compromised fetal growth resulting in intrauterine growth restriction (IUGR). Several studies have shown that IUGR offspring are susceptible to develop metabolic syndrome and CVDs, including ischemic heart disease, in later life (reviewed in (Alexander et al. 2015; Demicheva and Crispi 2014; Giussani and Davidge 2013)). More importantly, the cardiovascular health of a susceptible IUGR population has been shown to be affected by secondary stress factors such as a high‐fat (HF) diet (Rueda‐Clausen et al. 2012) or aging (Dasinger et al. 2016; Rueda‐Clausen et al. 2011).

Pregnancy complications are a unique situation in that fetal physiological phenomena frequently switch into a survival mode which is aimed at increasing oxygen delivery to vital fetal organs. Unfortunately, these adaptive changes at both a physiological and molecular level make the fetus more susceptible to a variety of diseases, including CVDs, in postnatal life. Therefore, clinical strategies involving pregnancy complications warrant the treatment and/or management of both immediate maternal and fetal health problems as well as their long‐term health concerns in later life. Resveratrol, a natural polyphenol found in grape skins, has been shown to have numerous cardiovascular effects. Scientific data from animal studies have shown the beneficial effect of resveratrol in the prevention of numerous CVDs such as cardiac hypertrophy (Chen et al. 2015; Dolinsky et al. 2015; Juric et al. 2007), hypertension (Care et al. 2016; Dolinsky et al. 2013), I/R injury (Hung et al. 2004; Shen et al. 2012), and heart failure (Raj et al. 2014; Zordoky et al. 2015) when treatment is given prior to the development of disease. Our laboratory has also shown the prevention of cardiovascular pathologies with resveratrol intervention in IUGR offspring fed a HF diet (Shah et al. 2015). Resveratrol‐mediated cardioprotection involves multiple signaling molecules such as activation of cardiac adenosine monophosphate kinase (AMPK) in rat (Gu et al. 2014), mitigation of mitochondrial reactive oxygen species production, upregulation of antioxidant enzymes such as superoxide dismutase (SOD) in cultured coronary artery endothelial cells (Ungvari et al. 2009) and aortic smooth muscle cells (Li et al. 2006). In addition, resveratrol‐mediated cardioprotection after I/R injury has been shown to involve mitigation of oxidative stress in rat hearts (Dernek et al. 2004; Ray et al. 1999).

While these animal studies have demonstrated that resveratrol prevented the development of a cardiac phenotype/pathology, only a few studies are available showing a reversal effect of resveratrol in the treatment of a cardiac pathology. For instance, treatment with resveratrol was able to reverse the cardiac pathology observed in mouse models of myocardial infarction (Kanamori et al. 2013) and pressure overload‐induced heart failure (Sung et al. 2015). However, there is a lack of sufficient evidence for a therapeutic effect of resveratrol in treating established disease and, further, the efficacy of resveratrol treatment in IUGR offspring whose CVD risk was programmed in fetal life has yet to be explored. Therefore, it is important to investigate the therapeutic effect of resveratrol to treat IUGR male and female offspring at the time of established cardiovascular dysfunction. It is of clinical relevance to know if resveratrol is a potential treatment option for the susceptible adult IUGR population presenting with preclinical signs of CVD. Therefore, we hypothesized that resveratrol treatment in later life, at a time of known CV dysfunction in adult IUGR offspring fed a HF diet, would improve both in vivo and ex vivo cardiac function. To determine the molecular mechanisms involved, we further investigated the effect of resveratrol on cardiac p‐AMPK levels and indices of cardiac oxidative stress; including cardiac antioxidant enzyme levels. Our findings will improve our understanding of the therapeutic role and effective timing of resveratrol intervention to ameliorate detrimental cardiovascular outcomes in a susceptible IUGR population in later life and identify potential molecular mechanisms involved.

## Materials and Methods

### Animal models

Female Sprague–Dawley rats weighing 250–275 g were obtained (Charles River, Quebec, Canada) and housed in a temperature controlled room with a 10:14‐h light–dark cycle. After acclimatization for 1 week, they were mated overnight and pregnancy was confirmed (day 0) by the presence of sperm in a vaginal smear obtained the next morning. Pregnant dams were fed standard chow [Lab Diet, Ref. 5001 (3.02 kcal/mg; protein 23%, fat 4.5%, fiber 6%)] throughout pregnancy. On day 15 of pregnancy, dams were randomly assigned to normoxia or maternal hypoxia protocols. Pregnant dams in the hypoxia protocol were individually housed in a plexiglass chamber where the oxygen concentration was maintained at 11% by the continuous infusion of nitrogen gas from gestational day 15–21. Both normoxic and hypoxic dams were allowed to give birth in a normal oxygen environment (21%). After birth, biometric measurements such as body weight, crown‐to‐rump length and abdominal girth were recorded and litters were culled to eight pups (4 males and 4 females) to control the postnatal environment. The fetal phenotype at birth was recently published elsewhere (Shah et al. 2015). In male and female offspring, prenatal hypoxia decreased the birth weight by 8.26% (*P *<* *0.05) and 8.45% (*P *<* *0.05), respectively. At 3 weeks of age, offspring were weaned and single housed. Out of each litter, two male and two female pups were utilized in a previous study (Shah et al. 2015) and the remaining pups were randomly assigned to receive either a HF diet (45% fat, D12451; Research Diet) for 18 weeks (from 3 to 21 weeks of age) or a HF diet (from 3 to 21 weeks of age) which was supplemented with resveratrol from 13 to 21 weeks of age (D120020402 4 g/kg; Research Diet). The dose of resveratrol was chosen based on previous studies (Dolinsky et al. 2011; Lagouge et al. 2006). Thus, the experimental groups consisted of: control‐HF, control‐HF + resveratrol, IUGR‐HF and IUGR‐HF + resveratrol. Offspring were randomized to experimental protocols such that no two animals in one group came from the same litter. Body weight and food intake were recorded once per week throughout the study duration. All procedures described in this study were approved by the University of Alberta Health Sciences Animal Policy and Welfare Committee and were in accordance with the guidelines of the Canadian Council on Animal Care. All experimental protocols conformed to the National Institutes of Health's Guide for the Care and Use of Laboratory Animals (eighth edition, Revised 2011).

### Echocardiography

At the age of 21 weeks, left ventricular (LV) morphology and function were assessed using echocardiography in male and female offspring as previously described (Ram et al. 2011). All echocardiographic assessments were done by a single operator. The echocardiography was performed in supine or semileft lateral decubitus position using a high‐resolution in vivo microimaging system Vevo 2100 (Visualsonics^®^, Toronto, ON, Canada) equipped with a 13–23 MHz linear array transducer (Ram et al. 2011). Animals were anesthetized (sedated with 4% isoflurane and 1 L/min compressed air and maintained at 1.5% isoflurane and 1 L/min compressed air) and echocardiography was performed in two‐dimensional guided M‐mode. The heart images were obtained in the two‐dimensional mode in the parasternal and short axis view with a depth of 2 cm. From this view, an M‐mode cursor was positioned perpendicular to the interventricular septum and posterior wall of the LV at the level of the papillary muscles. Parameters of LV morphology such as left ventricular internal diameter during diastole (LVID_d_) and systole (LVID_s_), diastolic and systolic interventricular septal wall thickness (IVS_d_ and IVS_s_), and diastolic and systolic left ventricular posterior wall thickness (LVPW_d_ and LVPW_s_) were obtained. Ejection fraction (EF) and fractional shortening (FS), were calculated using ventricular internal diameter, volume, and thickness obtained from M‐mode images according to the American Society of Echocardiography recommendations (Sahn et al. 1978), as follows:

EF(%)=[(LVEDV−LVESV)/LVEDV]∗100

$$\mathrm{FS}\left(\%\right)=[\left({\mathrm{LVID}}_{d}-{\mathrm{LVID}}_{s}\right)/{\mathrm{LVID}}_{d}]*100$$

where LVEDV is LV end diastolic volume and LVESV is LV end systolic volume.

In the apical four‐chamber view, E‐wave and A‐wave velocities, isovolumic relaxation time, isovolumic contraction time, E/A ratio, and deceleration time of E wave were obtained. Tissue Doppler imaging was used to obtain mitral annular velocities (E’ and A’).

### Isolated working heart preparation

Isolated working hearts were prepared using a previously described method (Rueda‐Clausen et al. 2009; Shah et al. 2015). At the age of 21 weeks (3–4 days after echocardiography), the heart was rapidly excised from anesthetized rats, kept in ice‐cold modified Krebs–Henseleit solution [120 mmol/L NaCl, 25 mmol/L NaHCO_3_, 5.5 mmol/L glucose, 4.7 mmol/L KCl, 1.2 mmol/L KH_2_PO_4_, 1.2 mmol/L MgSO_4_ and 2.5 mmol/L CaCl_2_ (pH 7.4)], inserted onto a cannula and the aorta was ligated with silk. Hearts were perfused with oxygenated Krebs–Henseleit solution for <10 min in retrograde Langendorff mode. During this time, excess pericardial tissues were removed and the left atrium was cannulated. After stabilization, hearts were perfused in anterograde working mode for 30 min (preischemia period) followed by 10 min of global ischemia (mild ischemia) and 40 min of reperfusion. Cardiac function was reported as cardiac power, calculated as: [(peak systolic pressure, mmHg – maximal preload, mmHg) x cardiac output, mL/min × 0.13]/dry weight, g] (joules/min/g dry wt). Cardiac parameters were recorded using an HSE data acquisition system and Isoheart software for Windows 2000 (Harvard Apparatus, Canada). Cardiac power was calculated every 10 min for a period of 80 min. Hearts which experienced nonreversible arrthymia were excluded from the study. Heart and left ventricle were weighed at the end of the experiment.

### Histological analysis

LV tissues were fixed in 10% neutral‐buffered formalin and embedded in paraffin. The LV tissue sections (5 *μ*m in thickness) were rehydrated with ethanol, stained with hematoxylin and eosin, dehydrated, and mounted on slides. Briefly, tissue was deparaffinized with xylene three times for 5 min, rehydrated with ethanol (100% ethanol two times for 2 min, 95% ethanol for 2 min, 70% ethanol for 1 min), and then washed with tap water for 2 min. Sections were treated with acid alcohol (70%) for 3 sec, washed with tap water for 1 min, 1% lithium carbonate added for 30 sec, and washed with tap water for 1 min. After dipping in 95% ethanol for 30 sec, tissues were incubated with Eosin Y for 33 sec, dehydrated and mounted on slides. H/E‐stained slides were examined under a light microscope (Evos xl core, ThermoFisher Scientific) and images were taken at 200× magnification. To measure diameter, only myocytes with a centrally located nucleus were included to ensure that the sections were in a perpendicular orientation. The myocyte diameter was obtained using image analysis software (Cell Sense, Olympus, Japan).

### DHE staining

Dihydroethidium (DHE) fluorescence was utilized to assess evidence of superoxide anions in cardiac tissue after I/R injury as previously described (Zanetti et al. 2005). LV tissue subjected to I/R injury was embedded in OCT and sliced (10 *μ*m thickness) using a cryostat. Sections were thawed, washed three times with Hank's balanced salt solution (HBSS), and incubated with DHE (200 *μ*mol/L) for 30 min at 37°C. Images were obtained using a fluorescence microscope (IX81 Olympus, Japan) with a 546 nm filter attached to an image analysis system (Cell Sense, Olympus, Japan). Fluorescent intensity was quantified using Image J software (Image J 1.48).

### Western blot analysis

At 21 weeks of age, LV tissue was collected at the end of working heart experiments, snap frozen and stored at −80°C until further analysis. Frozen tissues were homogenized in lysis buffer [in mmol/L: 20 Tris (pH 7.4), 5 EDTA, 10 Na_4_O_7_P_2_ sodium pyrophosphate tetrabasic, 100 sodium, 9 fluoride, and 1% NP‐40] containing protease (Protease Inhibitor Cocktail (1× Halt TM 200 protease inhibitor, 201 Thermo scientific) and 1 mmol/L PMSF, Fluka Biochemika) and phosphatase inhibitors (2 mmol/L Sodium Orthovanadate, Sigma). The protein concentration of the lysate was determined using a bicinchoninic acid assay (Pierce). A total of 100 *μ*g of protein was loaded and separated by SDS‐PAGE on a 7.5% or 10% polyacramide gel and transferred to a nitrocellulose membrane. The membrane was incubated with 50% blocking reagent for 1 h at room temperature. After washing with phosphate‐buffered saline solution, the membrane was incubated overnight at 4°C with primary antibodies for p‐AMPK (1:2000, Cell Signaling), SOD1 (1:1000, Santa Cruz Biochemicals), SOD2 (1:1000, Santa Cruz Biochemicals), Catalase (1:1000, Santa Cruz Biochemicals) or *β*‐actin (1:1000; Santa Cruz Biochemicals). The membrane was incubated with secondary antibody conjugated with fluorescent tag and blots were visualized with Li‐cor Odyssey Bioimager and quantified by densitometry with Odyssey V3.0 software (Li‐cor Biosciences).

### Statistical analysis

Data are presented as mean ± standard error of the mean and analyzed using GraphPad Prism 6 software. Statistical significance of differences among the four treatment groups was assessed using a two‐way ANOVA with IUGR and resveratrol as sources of variation, followed by a Bonferroni multiple comparison post test. A *P* ˂ 0.05 was considered statistically significant.

## Results

### Effect of resveratrol on body weight gain and food intake

**Figure 1:**
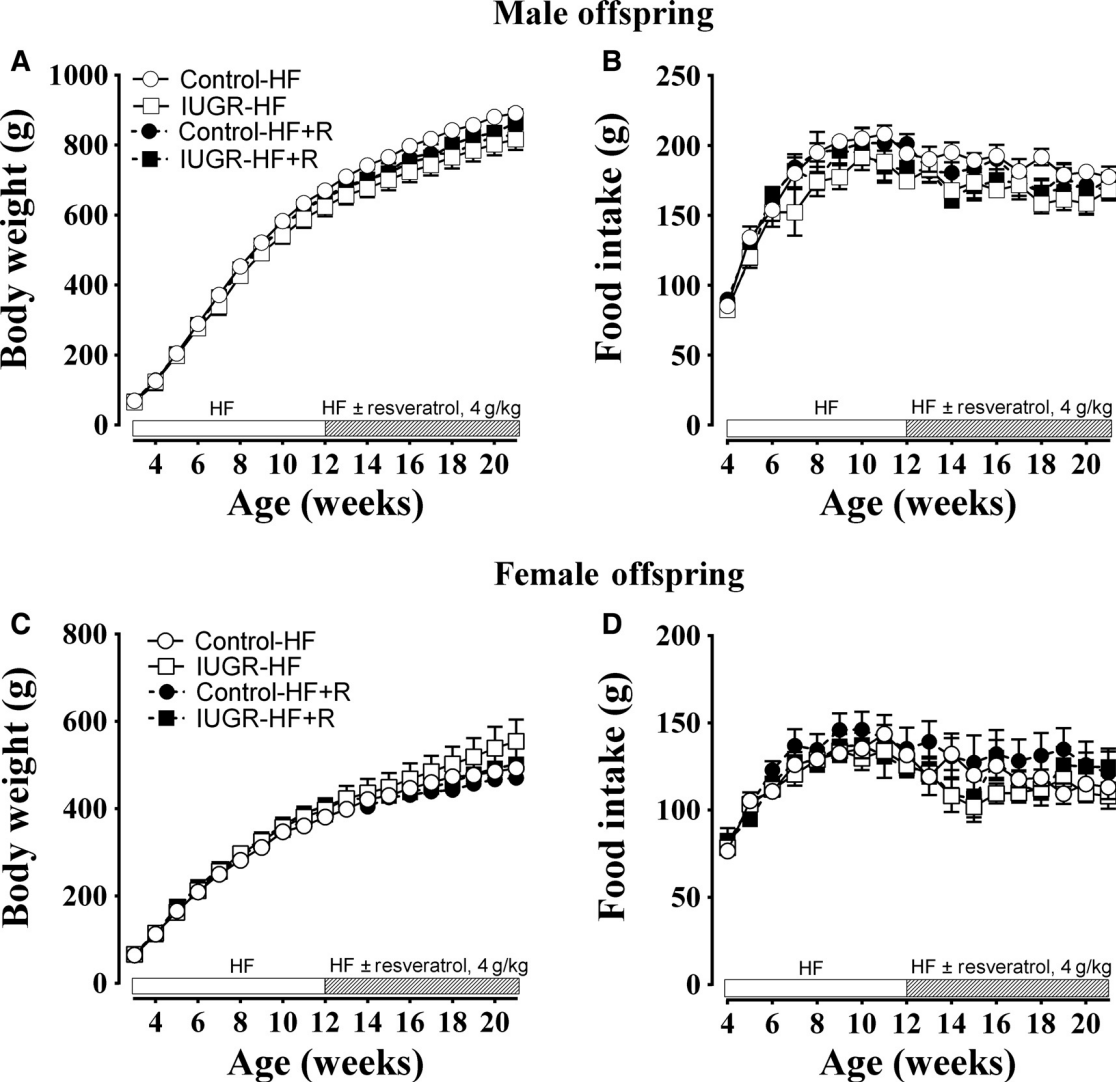
Body weight gain and food intake. Body weight (A. male and C. female) and food intake (B. male and D. female) were recorded from 3 to 21 weeks of age once/week in male offspring (from 9 to 10 dams) and female offspring (from 8 to 9 dams). All groups were compared using a two‐way ANOVA followed by a Bonferroni post hoc analysis. HF, high‐fat; R, resveratrol; IUGR, intrauterine growth restriction.

Prenatal hypoxia did not affect the body weight gain in male (Fig. 1A) or female (Fig 1C) offspring over the 18 week period in which they were fed on a HF diet. Furthermore, resveratrol supplementation in the diet did not affect the body weight gain in any group through this period (Fig. 1A and C). However, in females, an overall effect of increased body weight in IUGR offspring at 21 weeks of age was observed (Table 1). Food intake was not altered in either male (Fig. 1B) or female (Fig. 1D) offspring, among the groups throughout the intervention period, nor was food intake modified by resveratrol supplementation in the diet.

**Table 1:** Echocardiographic measurements in control and IUGR offspring at 21 weeks of age

	Control		IUGR				
Measurement	HF diet	HF diet + R	HF diet	HF diet + R	IUGR	Diet	Int
Male offspring							
Body weight, g	923.4 ± 30.8	932.5 ± 75.4	824.8 ± 49.6	946.8 ± 47.0			
Basal heart rate, beats/min	364 ± 14	363 ± 9	349 ± 9	334 ± 11.0			
Cardiac morphometry							
IVSd, mm	2.67 ± 0.31	2.18 ± 0.15	2.32 ± 0.07	2.28 ± 0.07			
IVSs, mm	4.81 ± 0.40	4.18 ± 0.08	4.28 ± 0.23	4.55 ± 0.18			
LVIDd, mm	9.40 ± 0.64	9.10 ± 0.33	9.08 ± 0.30	8.34 ± 0.27			
*LVIDs, mm*	**3.99 ± 0.3**	**4.32 ± 0.29**	**4.32 ± 0.2**	**3.31 ± 0.22**			*
LVPWd, mm	2.49 ± 0.10	2.28 ± 0.13	2.33 ± 0.11	2.40 ± 0.09			
LVPWs, m	**4.18 ± 0.23**	**3.89 ± 0.15**	**3.99 ± 0.15**	**4.43 ± 0.26**			*
Female offspring							
Body weight, g	**479.5 ± 24.4**	**456.8 ± 16.3**	**622.1 ± 46.1**	**523.5 ± 42.8**	*		
Basal heart rate, beats/min	393 ± 11	380 ± 18.6	374 ± 7	357 ± 21.2			
Cardiac morphometry							
IVSd, mm	1.88 ± 0.07	1.95 ± 0.14	2.09 ± 0.18	1.81 ± 0.13			
IVSs, mm	3.11 ± 0.23	3.52 ± 0.26	3.49 ± 0.16	2.93 ± 0.27			
LVIDd, mm	6.72 ± 0.46	6.51 ± 0.24	6.85 ± 0.20	7.19 ± 0.14			
LVIDs, mm	3.36 ± 0.3	2.90 ± 0.24	3.28 ± 0.3	3.68 ± 0.38			
LVPWd, mm	1.95 ± 0.05	2.09 ± 0.09	2.06 ± 0.16	1.80 ± 0.08			
LVPWs, mm	3.44 ± 0.25	3.58 ± 0.06	2.84 ± 0.36	3.22 ± 0.14			

Values are expressed as means ± SEM (*n *=* *5 per group). All groups were compared using a two‐way ANOVA followed by a Bonferroni post hoc test.

HF, high‐fat; R, resveratrol; IUGR, intrauterine growth restriction; Int, interaction; IVS, interventricular septum; LVID, left ventricle internal diameter; LVPW, left ventricular posterior wall; d, diastole; s, systole.

*P <* *0.05, for differences in the main effect (IUGR or resveratrol) or their interaction.

Bold indicates parameters that have significant effect of source of variation (IUGR or Resv) or their interaction.

### Effect of resveratrol on cardiac morphometry

**Figure 2:**
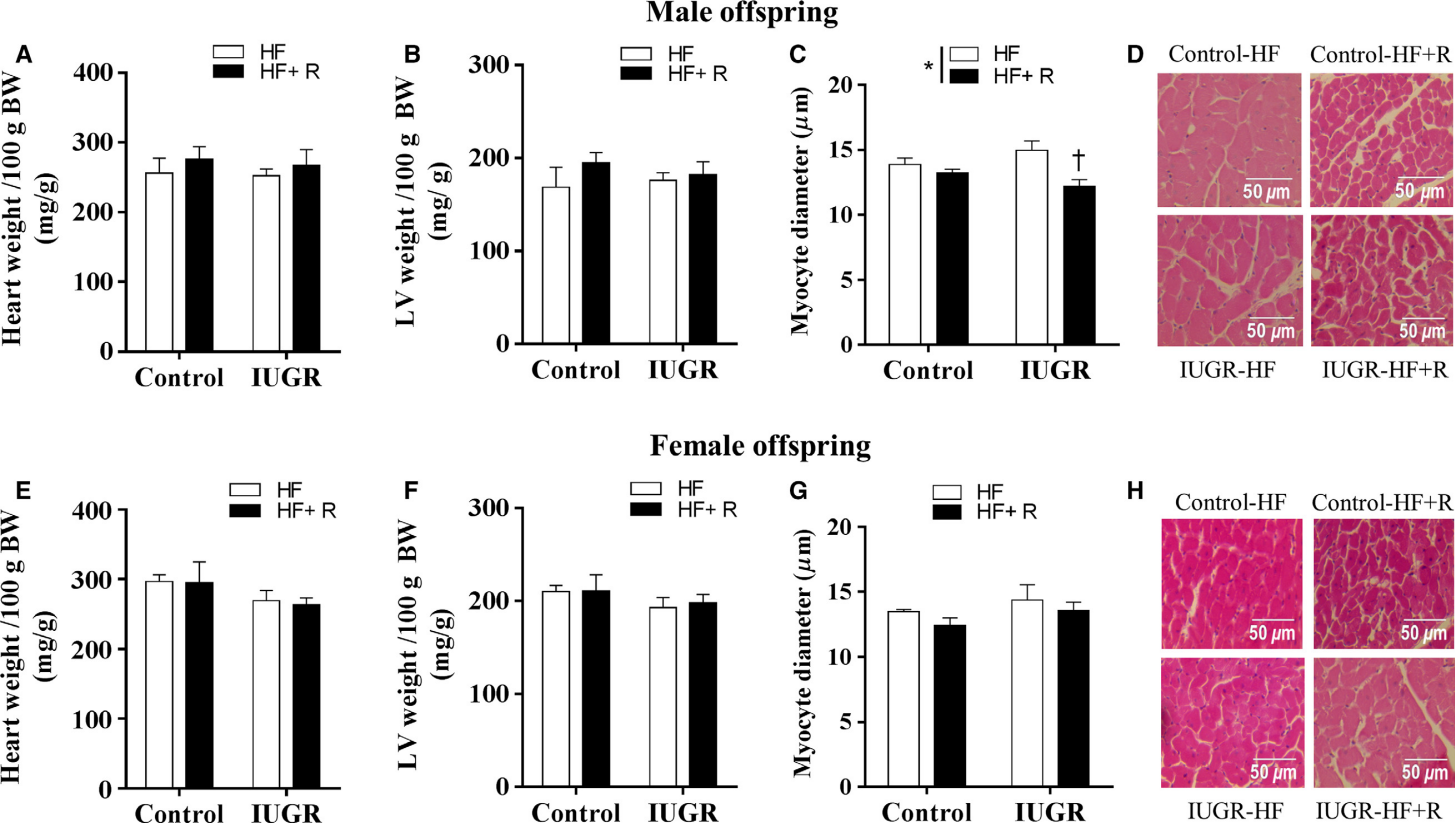
Ex vivo cardiac morphometry. In male and female offspring, the heart weight (A. male and E. female, *n *=* *7–9) and LV weight (B. male, *n *=* *7–9; F. female, *n *=* *7–8) were recorded at the end of I/R protocol at 21 weeks of age. Myocyte diameter was measured using histological images of LV stained with hematoxylin and eosin (C. male and G. female, *n *=* *3–5); representative images are shown (D. male and H. female). All groups were compared using a two‐way ANOVA followed by a Bonferroni post hoc test. *P <* *0.05, for differences in the main effect (IUGR or resveratrol). ^†^ *P *<* *0.05 versus IUGR‐HF; HF, high‐fat; R, resveratrol; IUGR, intrauterine growth restriction; LV, left ventricle.

Consistent with our previous findings in young adult offspring (3–4 months of age) (Rueda‐Clausen et al. 2009; Shah et al. 2015), our in vivo echocardiographic data showed that cardiac morphometry (LV septal and posterior wall thickness and LV internal diameter) was unaltered by prenatal hypoxia in male or female (Table 1) offspring at 21 weeks of age. There was an interaction effect on LV end systolic internal diameter and LV end systolic posterior wall thickness with opposing effects of resveratrol in control versus IUGR offspring (Table 1, *P *<* *0.05). In agreement with the in vivo data, ex vivo data revealed that there were no significant differences in heart weight or left ventricular weight in either male (Fig. 2A–B) or female (Fig. 2E–F) offspring.

At the cellular level, cardiac myocyte diameter was assessed using histological analysis. In male offspring (Fig. 2C–D), H/E staining of cardiac tissue sections showed that there was an overall effect of resveratrol treatment in decreasing cardiac myocyte diameter; which was significant in IUGR offspring. However, in female offspring (Fig. 2G–H), there was no significant difference in cardiac myocyte diameter among the groups; independent of IUGR or resveratrol treatment.

### Effect of resveratrol on in vivo cardiac function

In male and female offspring (Table 2), systolic function parameters such as cardiac output and LV shortening fraction were not affected by IUGR. However, in only male offspring there was an interaction effect on LV ejection fraction with opposing effects of resveratrol in control versus IUGR offspring (*P *<* *0.05).

**Table 2:** Systolic function in control and IUGR offspring at 21 weeks of age

	Control		IUGR				
Measurement	HF Diet	HF Diet + R	HF Diet	HF Diet + R	IUGR	Diet	Int
Male offspring							
LVEDV, *μ*L	454.2 ± 44	457.8 ± 33.8	452.4 ± 37.2	370.3 ± 25.1			
LVESV, *μ*L	68.3 ± 11.4	86.7 ± 14.4	84.7 ± 11.2	42.1 ± 8.1			
CO, mL/min	138.4 ± 12.2	125.8 ± 6.9	125.9 ± 10.4	109.7 ± 8.1			
EF, %	**84.4 ± 3.0**	**81.4 ± 2.5**	**81.0 ± 2.7**	**88.8 ± 1.6**			*
FS, %	56.4 ± 3.7	52.3 ± 2.5	52.2 ± 2.8	61.5 ± 2.3			
Female offspring							
LVEDV, *μ*L	284.7 ± 30.0	252.6 ± 11.8	264.7 ± 19.6	296.3 ± 16.1			
LVESV, *μ*L	37 ± 6.2	28.2 ± 3.6	37.4 ± 10	42.5 ± 10			
CO, mL/min	97 ± 9.6	86.3 ± 9.6	84.7 ± 5.1	91.5 ± 10.5			
EF, %	87.1 ± 1.5	88.6 ± 1.7	86 ± 3.1	85.8 ± 3.1			
FS, %	58.6 ± 1.9	61 ± 2.9	57.5 ± 3.7	57.5 ± 4.0			

Values are expressed as means ± SEM (*n *=* *5 per group). All groups were compared using a two‐way ANOVA followed by a Bonferroni post hoc test.

HF, high‐fat; R, resveratrol; IUGR, intrauterine growth restriction; Int, interaction; LVEDV, left ventricle end diastolic volume; LVESV, left ventricle end systolic volume; CO, cardiac output; EF, ejection fraction; FS, fractional shortening.

*P <* *0.05, for interaction between two variations (IUGR or resveratrol).

Bold indicates parameters that have significant effect of source of variation (IUGR or Resv) or their interaction.

In male IUGR offspring (Table 3), diastolic function parameters such as mitral E‐wave velocity, A‐wave velocity, deceleration time, and isovolumic relaxation time were altered as compared to control offspring. E‐ and A‐wave velocities were significantly decreased in male IUGR offspring without altering the E/A ratio while deceleration time and isovolumic relaxation time were significantly increased as compared to controls; suggesting an established diastolic dysfunction (Table 3). In contrast, diastolic function parameters (E wave, A wave, and E/A ratio) were not altered in female IUGR offspring compared to their control counterparts. However, isovolumic relaxation time was significantly longer in female IUGR offspring (Table 3) and there was an interaction effect for deceleration time indicating an early sign of diastolic dysfunction. Interestingly, and contrary to our expectation, resveratrol treatment did not improve any signs of diastolic dysfunction observed in either male or female IUGR offspring (Table 3).

**Table 3:** Diastolic function in control and IUGR offspring at 21 weeks of age

	Control		IUGR				
Measurement	HF Diet	HF Diet + R	HF Diet	HF Diet + R	IUGR	Diet	Int
Male offspring							
E, mm/s	**1026.1 ± 51.2**	**1021 ± 14.3**	**898.9 ± 44.1**	**810.9 ± 56.9**	**		
A, mm/sec	**904.7 ± 49.0**	**926.2 ± 37.4**	**757.5 ± 59.8**	**740.5 ± 71.0**	**		
E/A	1.11 ± 0.01	1.07 ± 0.02	1.22 ± 0.12	1.10 ± 0.04			
DT, msec	**34.6 ± 3.6**	**43.5 ± 1.7**	**45.7 ± 3.8**	**47.6 ± 1.6**	*		
IVRT, msec	**22.4 ± 1.0**	**20.5 ± 0.8**	**25.3 ± 0.5**	**26.0 ± 1.5**	***		
MPI	0.66 ± 0.02	0.62 ± 0.03	0.69 ± 0.02	0.74 ± 0.06			
E’, mm/sec	40.8 ± 3.9	39.9 ± 4.1	35.2 ± 4.4	35.2 ± 2.3			
A’, mm/sec	44.7 ± 4.5	41 ± 4.4	40.3 ± 5.0	37.8 ± 3.3			
E’/A’	0.92 ± 0.03	0.98 ± 0.01	0.88 ± 0.04	0.95 ± 0.06			
E/E’	25.7 ± 1.5	26.9 ± 3.4	28.8 ± 6.8	23.4 ± 2.5			
Female offspring							
E, mm/sec	901.1 ± 83.0	906.1 ± 47.3	972.7 ± 75.4	788.2 ± 52.5			
A, mm/sec	684.9 ± 94.8	786.1 ± 40.9	756.2 ± 101	663.8 ± 61.7			
E/A index	1.36 ± 0.1	1.16 ± 0.04	1.32 ± 0.1	1.20 ± 0.09			
DT, msec	**45.3 ± 1.3**	**39.5 ± 1.0**	**40.0 ± 2.8**	**43.1 ± 2.5**			*
IVRT, msec	**23.0 ± 1.0**	**22.6 ± 0.5**	**24.3 ± 1.5**	**26.2 ± 1.2**	*		
MPI	0.69 ± 0.02	0.74 ± 0.03	0.65 ± 0.05	0.68 ± 0.04			
E’, mm/sec	36.4 ± 2	35.7 ± 4.8	36 ± 2.6	37.1 ± 5.7			
A’, mm/sec	38.4 ± 2.7	37.7 ± 4.0	40.1 ± 4.0	36.2 ± 3.8			
E’/A’	0.96 ± 0.05	0.94 ± 0.04	0.91 ± 0.04	0.97 ± 0.07			
E/E’	25.3 ± 3.3	27.3 ± 3.8	27.4 ± 2.9	22.5 ± 2.8			

Values are expressed as mean ± SEM (*n* = 5 per group). All groups were compared using a two‐way ANOVA followed by a Bonferroni post hoc test.

HF, high‐fat; R, resveratrol; IUGR, intrauterine growth restriction; Int, interaction; E, peak velocity of early mitral inflow; A, peak velocity of late mitral inflow; DT, deceleration time; IVRT, isovolumic relaxation time; MPI, myocardial performance index; E’, early diastolic velocity of mitral annulus; A’, late diastolic velocity of mitral annulus.

*P < 0.05, **P < 0.01, ***P < 0.001 for differences in the main effect (IUGR or resveratrol) or their interaction.

Bold indicates parameters that have significant effect of source of variation (IUGR or Resv) or their interaction.

### Effect of resveratrol on ex vivo cardiac function

**Figure 3:**
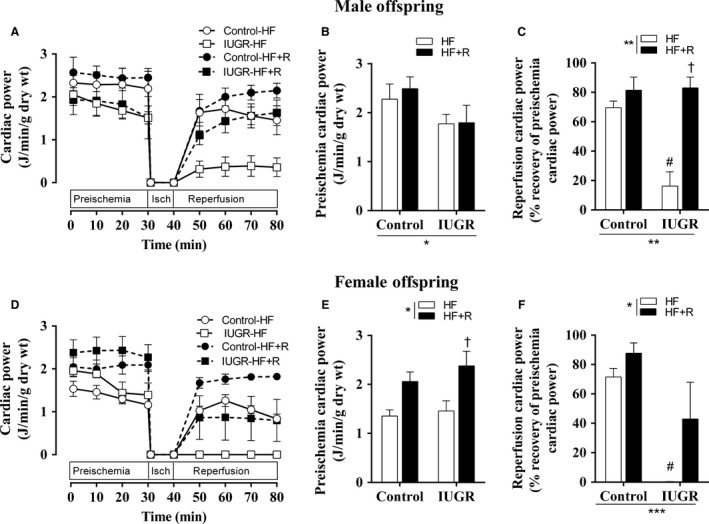
Ex vivo cardiac function during I/R protocol. Cardiac power (A. male and D. female) was recorded in isolated perfused hearts using a Langendorff heart system. After stabilization, preischemia cardiac power was measured for 30 min as a baseline. Following 10 min of global ischemia, reperfusion cardiac power was measured for 40 min in male and female offspring. Absolute preischemia cardiac power and percent change in reperfusion cardiac power after I/R are shown in male (B and C) and female (E and F) offspring. All groups were compared using a two‐way ANOVA followed by a Bonferroni post hoc test (*n *=* *4–5, male and *n *=* *4–6, female). *P <* *0.05, **P <* *0.01, ***P <* *0.001 for differences in the main effect (IUGR or resveratrol), #*P *<* *0.05 versus control‐HF, †*P *<* *0.05 versus IUGR‐HF. HF, high‐fat; R, resveratrol; IUGR, intrauterine growth restriction; I/R, ischemia/reperfusion.

The ability of the heart to recover after a mild I/R injury was assessed in male and female offspring. In male offspring (Fig. 3A–B), an overall effect of IUGR was observed with IUGR offspring demonstrating a lower baseline cardiac power. However, the baseline cardiac power within the control or IUGR groups was not affected by resveratrol treatment (Fig. 3B). The cardiac power recovery after mild I/R injury was comparable within the control groups with or without resveratrol treatment (Fig. 3C). In contrast, offspring born following IUGR demonstrated cardiac susceptibility to I/R injury as shown by a decrease in cardiac power recovery after exposure to mild ischemia (Fig. 3C). Interestingly, resveratrol treatment in later life was able to improve the cardiac function after I/R injury in male IUGR offspring (Fig. 3C).

In contrast to male offspring, there was an overall effect of resveratrol treatment on the baseline cardiac power in female offspring (Fig. 3D–E). Furthermore, resveratrol treatment significantly increased preischemia cardiac power in IUGR female offspring (Fig. 3E). An overall effect of both IUGR, decreasing recovery of cardiac power, and resveratrol treatment, increasing recovery of cardiac power, was observed during the recovery period in IUGR offspring (Fig. 3F). These data indicate that the effects of resveratrol on cardiac function recovery after I/R were evident in both sexes in our animal model.

### Effect of resveratrol on cardiac p‐AMPK levels

**Figure 4:**
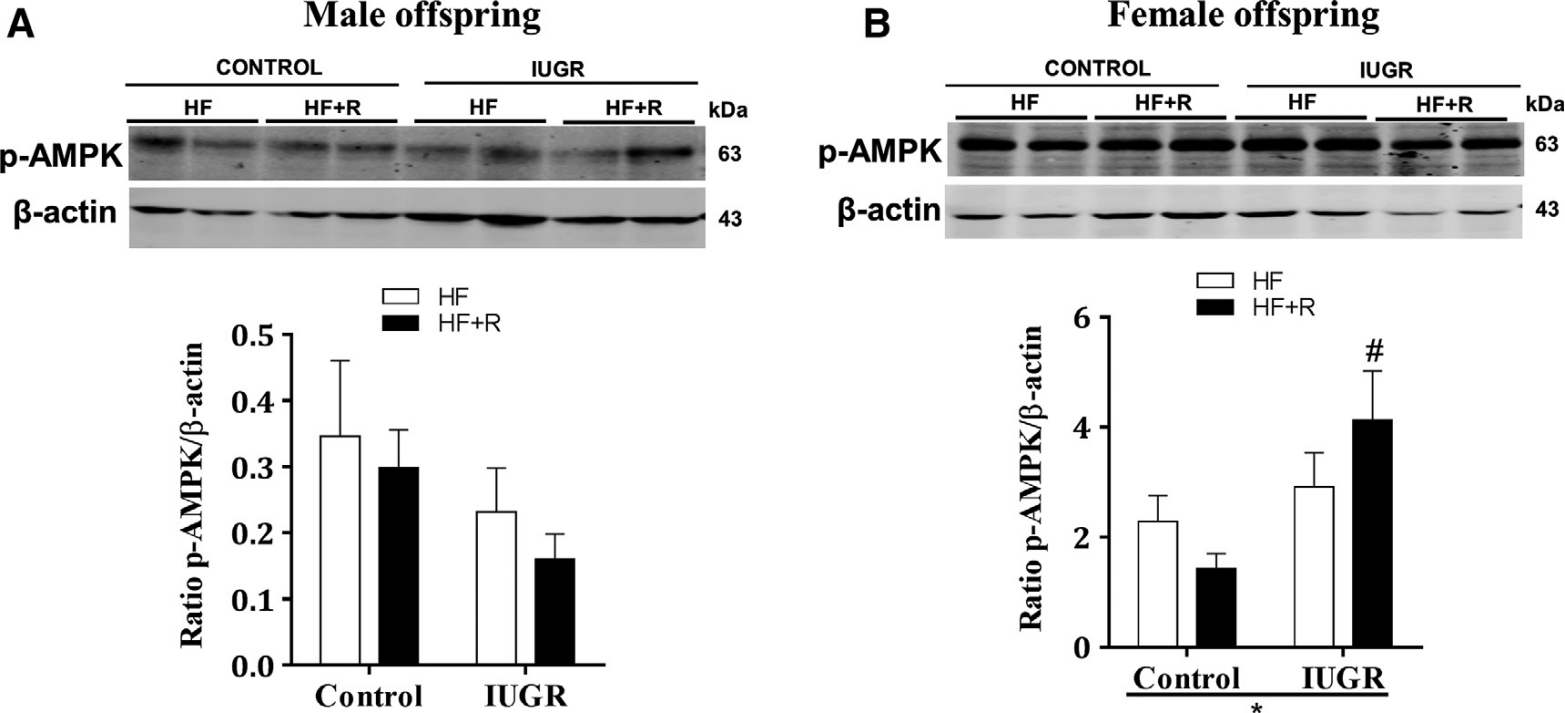
Western blot analysis for cardiac p‐AMPK protein levels. In male and female offspring, proteins were measured in heart tissue lysates after 18 weeks of dietary intervention. Blots were quantified using Odyssey software and expressed as p‐AMPK/*β*‐actin ratio (A. male and B. female). All groups were compared using a two‐way ANOVA followed by a Bonferroni post hoc test (male and female, *n *=* *4–5). *P <* *0.05 for differences in the main effect (IUGR or resveratrol), #*P *<* *0.05 vs. control‐HF + R. HF, high‐fat; R, resveratrol; IUGR, intrauterine growth restriction.

In male offspring (Fig. 4A), cardiac p‐AMPK protein levels were unaltered among the groups. Furthermore, resveratrol did not affect the cardiac p‐AMPK levels in male control or IUGR offspring (Fig. 4A). In female offspring, there was an overall effect of IUGR in increasing cardiac p‐AMPK levels and resveratrol treatment significantly increased cardiac p‐AMPK levels in only female IUGR offspring. In contrast, resveratrol treatment significantly increased cardiac p‐AMPK levels in female IUGR offspring (Fig. 4B).

### Effect of resveratrol on cardiac oxidative stress

**Figure 5:**
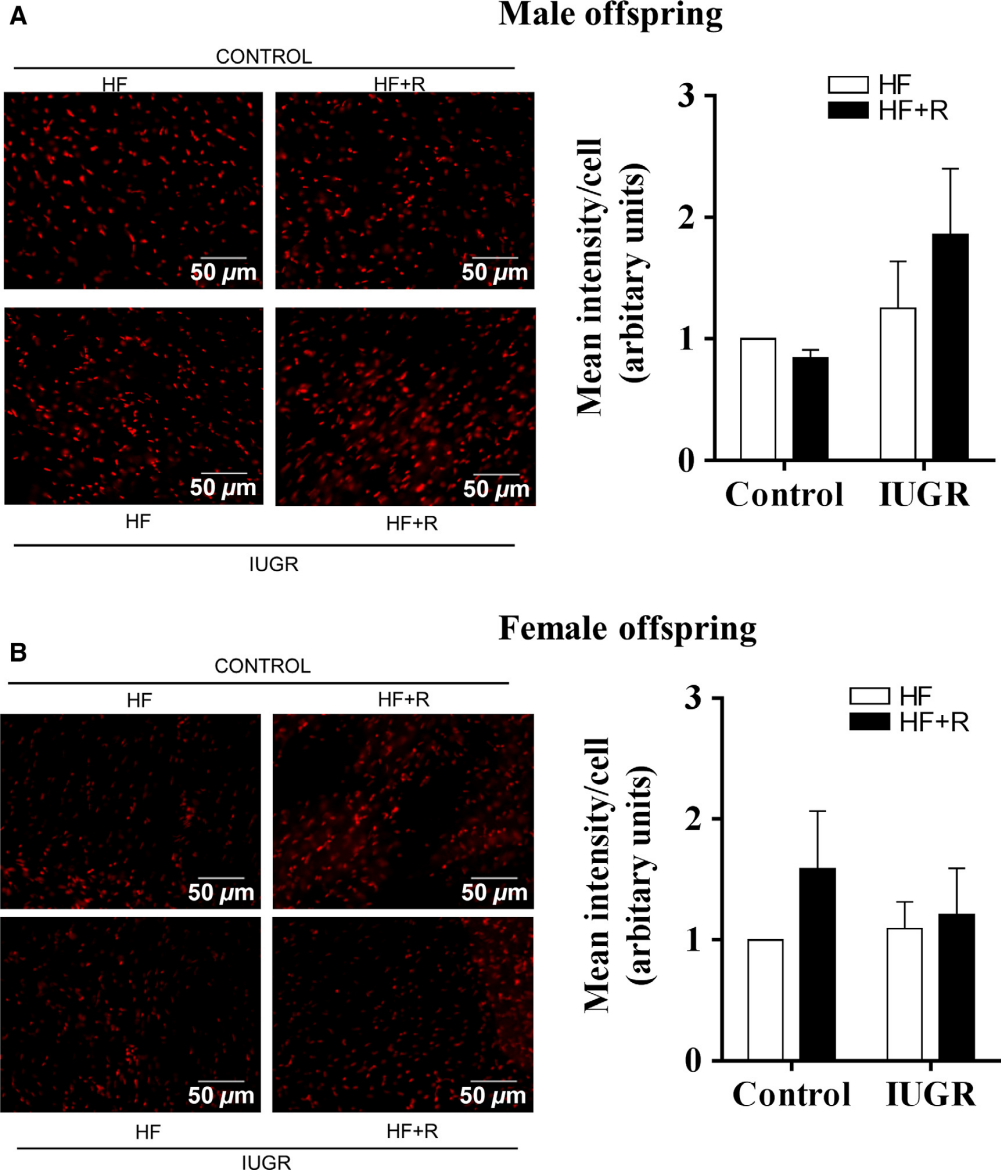
Cardiac superoxide levels in male and female offspring. Representative microscopic images of heart tissue sections stained with dihydroethidium in male (A) and female (B) offspring. Data were expressed as mean intensity/cell after quantification of images using fluorescence microscope. All groups were compared using a two‐way ANOVA followed by a Bonferroni post hoc test (male and female, *n *=* *4–5). HF, high‐fat; R, resveratrol; IUGR, intrauterine growth restriction.

**Figure 6:**
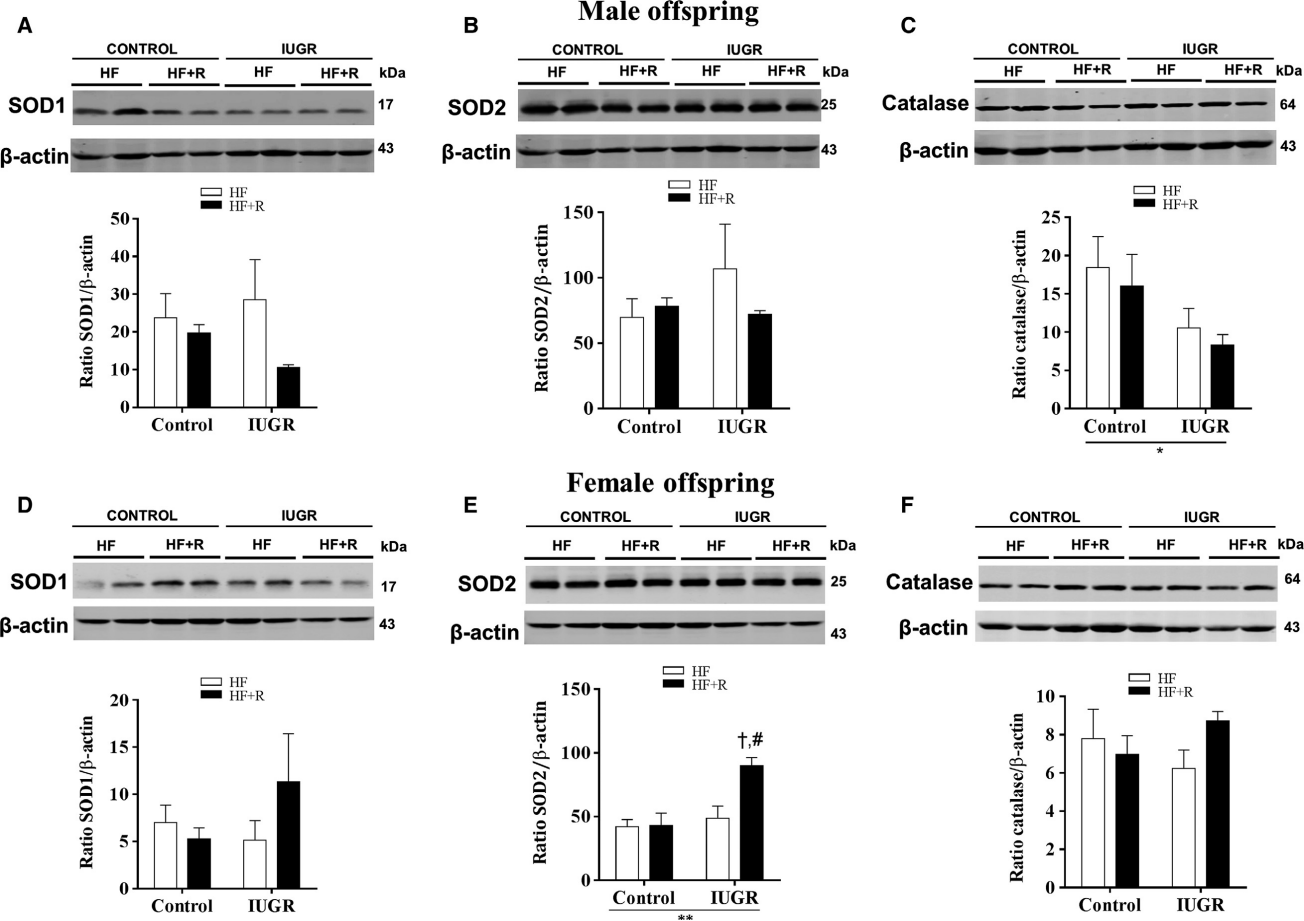
Western blot analysis for cardiac SOD1, SOD2, and catalase levels. In male and female offspring, antioxidants were measured in heart tissue lysates after 18 weeks of dietary intervention. Blots were quantified using Odyssey software and expressed as SOD1/*β*‐actin, SOD2/*β*‐actin or catalase/*β*‐actin ratios (A–C. male and D–F. female). All groups were compared using a two‐way ANOVA followed by a Bonferroni post hoc test (*n *=* *3–5 per group). Outliers identified using Grubbs’ test were not included in the analyses. **P <* *0.01 for differences in the main effect (IUGR or resveratrol), ^#^ *P *<* *0.05 versus control‐HF + R, ^†^ *P *<* *0.05 versus IUGR‐HF. HF, high‐fat; R, resveratrol; IUGR, intrauterine growth restriction; SOD, superoxide dismutase.

Prenatal hypoxia did not increase cardiac superoxide levels in either male (Fig 5A) or female (Fig. 5B) offspring. In addition, superoxide levels were unaffected by resveratrol treatment. Following this observation, we further evaluated the levels of the cardiac antioxidant enzymes SOD1, SOD2 and catalase. In male offspring, neither IUGR nor resveratrol treatment affected cardiac SOD1 or SOD2 levels (Fig. 6A and B). However, there was an overall effect of IUGR in decreasing cardiac catalase levels; which were not affected by resveratrol treatment (Fig. 6C). In female offspring, resveratrol significantly increased cardiac SOD2 levels (Fig. 6E), whereas neither SOD1 nor catalase were affected by IUGR or resveratrol treatment (Fig. 6D and F).

## Discussion

Epidemiological and experimental studies have shown that populations born following IUGR are more susceptible to developing long‐term health complications including CVDs. In the prenatal hypoxia rat model, cardiac dysfunction after I/R injury has been observed in adult IUGR offspring (3–4 months old) (Rueda‐Clausen et al. 2011; Xue and Zhang 2009). Furthermore, we have found that adult male and female IUGR offspring, in the presence of postnatal HF exposure for 9 weeks (from 3 to 12 weeks of age), demonstrate exacerbated cardiac dysfunction after mild I/R injury with preserved in vivo cardiac function (Rueda‐Clausen et al. 2011; Shah et al. 2015). However, in vivo cardiac dysfunction, in particular diastolic dysfunction, was observed in IUGR offspring with aging (12 months of age) (Rueda‐Clausen et al. 2009). The interesting findings of this study are as follows: (1) Male IUGR offspring developed more pronounced signs of in vivo diastolic dysfunction, whereas female IUGR offspring had early signs of diastolic dysfunction, (2) As expected, compared to their controls, male and female IUGR offspring fed a HF diet for 18 weeks (3–21 weeks of age) were more susceptible to ex vivo cardiac dysfunction after I/R injury. However, interestingly, the relative cardioprotective effect against I/R injury previously observed in female IUGR offspring on a HF diet for a shorter (9 wks) duration (3–12 weeks of age) (Ray et al. 1999) was abolished, and (3) Resveratrol treatment of IUGR offspring at the time of established CV dysfunction reversed ex vivo cardiac dysfunction after I/R injury in both male and female IUGR offspring without affecting in vivo cardiac diastolic dysfunction. Taken together, the data suggest that CV dysfunction programmed in utero due to prenatal hypoxia results in the clinical manifestation of in vivo cardiac diastolic dysfunction and greater cardiac susceptibility to I/R injury after chronic postnatal HF exposure in both male and female offspring. Despite having an equal cardiac susceptibility to I/R injury, female IUGR offspring exhibited greater tolerance to in vivo CV dysfunction. Interestingly, resveratrol treatment following established CV dysfunction was able to reverse the greater susceptibility of ex vivo cardiac dysfunction observed in IUGR offspring after I/R injury, independent of sex, signifying a therapeutic role of resveratrol in treating cardiovascular pathologies programmed in utero that manifest in an ischemic cardiac condition in the clinical setting.

CVD is associated with several risk factors such as sex, age, genetics, and prenatal hypoxia. We and others have shown that prenatal hypoxia during pregnancy poses a risk to offspring for long‐term adverse outcomes including cardiovascular health (reviewed in (Alexander et al. 2015; Demicheva and Crispi 2014; Giussani and Davidge 2013)). Studies have shown that a susceptible population which experiences prenatal hypoxia may develop clinical manifestations of cardiac disease in early life with exposure to secondary postnatal stress factors (Rueda‐Clausen et al. 2012; Shah et al. 2015); suggesting that IUGR offspring may have a subclinical phase of cardiovascular dysfunction in adult life. In fact, in many instances, CVDs may undergo a subclinical phase for decades before the first clinical manifestation appears (Berenson 2002). Interestingly, the severity of cardiovascular risk due to fetal programming, as shown in animal models, is sex‐specific (reviewed in (Dasinger and Alexander 2016)). In reduced uterine perfusion pressure and maternal low protein rat models, it was observed that male IUGR offspring had increased blood pressure in young adulthood compared to female IUGR offspring (Alexander 2003; Woods et al. 2001); an effect that might be associated with an alteration in components of the renin angiotensin system (Ojeda et al. 2007). Furthermore, ex vivo experiments using left ventricular bundles from adult IUGR offspring born to nutrient restricted dams showed contractile dysfunction, impaired calcium handling and decreased in RYR2 protein levels in males, but not females (Harvey et al. 2015). In this study, at 5 months of age male IUGR offspring had mild diastolic dysfunction (stage 1 diastolic dysfunction in human cardiac diseases (Gabriel and Klein 2009), as indicated by increased deceleration time and isovolumic relaxation time. In contrast, in female IUGR offspring, changes in diastolic function were not as evident; the only change observed was a prolonged isovolumic relaxation time indicating an early sign of diastolic dysfunction. However, our previous studies have shown that both male and female IUGR offspring develop a severe diastolic pathology at 12 months of age (Rueda‐Clausen et al. 2009). Based on these findings, it is possible that female IUGR offspring are somewhat cardioprotected at an early stage of life or, at least, the development of diastolic cardiac pathology is delayed in females and becomes more pronounced with aging (Rueda‐Clausen et al. 2009). Moreover, in these 12 month old IUGR offspring, there is an increased E/A ratio and decreased deceleration time and isovolumic relaxation time; echocardiographic findings similar to stage 3 or stage 4 diastolic dysfunction in human cardiac diseases (Gabriel and Klein 2009) which reflects a progression of cardiac diastolic dysfunction to an advanced stage in both male and female IUGR offspring with aging.

This study showed decreased cardiac power recovery after I/R injury in both male and female IUGR offspring compared to their control counterparts which, although expected, is interesting when considering the sexual dimorphism in the susceptibility to I/R injury that was observed in the previous study; females exhibiting better tolerance to I/R injury (Shah et al. 2015). Of note, this discrepancy could be attributed to the differences in duration of postnatal HF exposure (9 weeks vs. 18 weeks HF exposure) and age (12 weeks vs. 21 weeks). Thus the finding implicates that the chronic exposure of a susceptible IUGR population to postnatal stress factors exacerbates the manifestation of CVDs independent of sex and reflects the varying degree of susceptibility depending on compounding factors and postnatal environments.

Resveratrol, a polyphenol found in grape skins, has been demonstrated to have multiple cardio‐beneficial effects including antihypertension, antihypertrophy and protection against I/R injury (Zordoky et al. 2015). Consistent with our previous findings (Shah et al. 2015), we observed that neither prenatal hypoxia nor resveratrol significantly affected gross cardiac morphometry in male or female offspring. However, resveratrol decreased the myocyte diameter in male IUGR offspring indicating an antihypertrophic effect at a cellular level. Using isolated perfused hearts, it has been shown that resveratrol administration in the perfusate before global ischemia or during reperfusion improved the cardiac function recovery after I/R injury (Das et al. 2005; Goh et al. 2007). In line with these reports, we have previously demonstrated that dietary supplementation of resveratrol in early life prevented cardiac dysfunction after I/R injury in male and female IUGR offspring fed a HF diet (Rueda‐Clausen et al. 2012; Shah et al. 2015). However, there are few studies available that have shown the therapeutic effects of resveratrol in treating CVDs. For instance, resveratrol treatment has only been shown to reverse the cardiac pathology observed in mouse models of myocardial infarction (Kanamori et al. 2013) and pressure overload‐induced heart failure (Sung et al. 2015). In agreement with these studies, our study also showed that resveratrol treatment of IUGR offspring at the time of established CV dysfunction improved ex vivo cardiac function recovery after I/R injury in male and female IUGR offspring. However, we also demonstrated that resveratrol treatment did not improve the observed in vivo cardiac diastolic dysfunction; suggesting that there may be a window of opportunity for treatment in our animal models.

The cardiovascular effects of resveratrol have been shown to involve multiple molecular targets (reviewed in (Li et al. 2012)). In an H9c2 cardiac cell culture model, it has been shown that resveratrol prevented cardiac injury through AMPK activation in a condition that mimics I/R injury (Hwang et al. 2008). In addition, resveratrol‐mediated cardioprotection after I/R injury has been shown to involve mitigation of oxidative stress in rat hearts (Dernek et al. 2004; Ray et al. 1999) and increase antioxidant enzymes such as SOD activity in neonatal cardiomyocytes (Shen et al. 2012). It has also been shown that resveratrol can prevent cardiac dysfunction via AMPK activation in a rat myocardial infarction/heart failure model (Gu et al. 2014). Furthermore, resveratrol treatment for 2 weeks in male mice with established heart failure has been shown to reverse the cardiac pathology via up‐regulation of cardiac p‐AMPK and an increase in antioxidant levels (Kanamori et al. 2013; Sung et al. 2015). Therefore, we investigated cardiac p‐AMPK levels and antioxidant systems in cardiac tissue to identify potential mechanisms involved in the cardio‐beneficial effects of resveratrol. Prenatal hypoxia did not alter cardiac p‐AMPK levels in either male or female offspring. In contrast to other studies (Kanamori et al. 2013; Sung et al. 2015), resveratrol treatment also did not alter cardiac p‐AMPK levels in male IUGR offspring; which may be due to different resveratrol doses, timing of intervention or the animal models utilized in these studies. However, it is interesting to observe that resveratrol treatment increased cardiac p‐AMPK levels in female IUGR offspring; suggesting that this might be one of the mechanisms for the better in vivo cardiac diastolic function observed in female IUGR offspring compared to their male counterparts. However, this mechanism was not able to fully sustain diastolic function in female IUGR offspring. This finding suggests that in female IUGR offspring resveratrol may activate cardiac AMPK. In fact, it has been reported that resveratrol can activate AMPK directly (Sin et al. 2014) or via an upstream signaling molecule such as liver kinase B1(Dolinsky et al. 2009).

In contrast to our previous study (Shah et al. 2015), indices of oxidative stress were not increased in male or female IUGR offspring compared to controls. However, expected effects of prenatal environment might be masked due to the longer exposure to a HF diet (9 weeks vs. 18 weeks) and aging (3 months vs. 5 months of age); both of which are also known prooxidants. Resveratrol treatment did not modify cardiac superoxide levels in male or female offspring. Following this observation, we further assessed cardiac antioxidant enzymes. Interestingly, resveratrol increased cardiac SOD2 levels in only female but not male IUGR offspring. Taken together, these findings suggest that the potential mechanism of action for the cardio‐beneficial effects of resveratrol treatment may involve an increase in cardiac p‐AMPK and SOD2 levels in the susceptible female IUGR population.

## Conclusions

We demonstrated that resveratrol treatment of IUGR offspring, at a time of established CV dysfunction, improved ex vivo cardiac dysfunction after I/R injury in both male and female IUGR offspring. Thus, the findings of this study highlight the beneficial effects of resveratrol in the treatment of cardiac pathologies manifested in susceptible IUGR populations; specifically in a pathological condition where reperfusion‐induced cardiac injury is most commonly encountered such as ischemic heart diseases.

## Conflict of Interest

None declared.

## References

[phy213109-bib-0001] Alexander, B. T . 2003 Placental insufficiency leads to development of hypertension in growth‐restricted offspring. Hypertension (Dallas, Tex: 1979) 41: 457–462. 10.1161/01.HYP.0000053448.95913.3D 12623943

[phy213109-bib-0002] Alexander, B. T. , J. H. Dasinger , and S. Intapad . 2015 Fetal programming and cardiovascular pathology. Compr. Physiol. 5:997–1025. 25880521 10.1002/cphy.c140036 PMC4772789

[phy213109-bib-0003] Berenson, G. S. 2002 Childhood risk factors predict adult risk associated with subclinical cardiovascular disease. The Bogalusa Heart Study. Am. J. Cardiol. 90:3 l–7l. 10.1016/s0002-9149(02)02953-3 12459418

[phy213109-bib-0004] Care, A. S. , M. M. Sung , S. Panahi , F. S. Gragasin , J. R. Dyck , S. T. Davidge , et al. 2016 Perinatal resveratrol supplementation to spontaneously hypertensive rat dams mitigates the development of hypertension in adult offspring. Hypertension (Dallas, Tex: 1979) 67: 1038–1044. 10.1161/HYPERTENSIONAHA.115.06793 26928803

[phy213109-bib-0005] Chen, T. , J. Li , J. Liu , N. Li , S. Wang , H. Liu , et al. 2015 Activation of SIRT3 by resveratrol ameliorates cardiac fibrosis and improves cardiac function via the TGF‐beta/Smad3 pathway. Am. J. Physiol. Heart Circ. Physiol. 308:H424–H434. 25527776 10.1152/ajpheart.00454.2014

[phy213109-bib-0006] Das, S. , G. A. Cordis , N. Maulik , and D. K. Das . 2005 Pharmacological preconditioning with resveratrol: role of CREB‐dependent Bcl‐2 signaling via adenosine A3 receptor activation. Am. J. Physiol. Heart Circ. Physiol. 288:H328–H335. 15345477 10.1152/ajpheart.00453.2004

[phy213109-bib-0007] Dasinger, J. H. , and B. T. Alexander . 2016 Gender differences in developmental programming of cardiovascular diseases. Clin. Sci. (London, England: 1979) 130: 337–348. 10.1042/CS20150611 PMC4912835 26814204

[phy213109-bib-0008] Dasinger, J. H. , S. Intapad , M. A. Backstrom , A. J. Carter , and B. T. Alexander . 2016 Intrauterine growth restriction programs an accelerated age‐related increase in cardiovascular risk in male offspring. Am. J. Physiol. Renal. Physiol. 311:F312–F319. 27147668 10.1152/ajprenal.00123.2016 PMC5005278

[phy213109-bib-0009] Demicheva, E. , and F. Crispi . 2014 Long‐term follow‐up of intrauterine growth restriction: cardiovascular disorders. Fetal Diagn. Ther. 36:143–153. 23948759 10.1159/000353633

[phy213109-bib-0010] Dernek, S. , M. Ikizler , N. Erkasap , B. Ergun , T. Koken , K. Yilmaz , et al. 2004 Cardioprotection with resveratrol pretreatment: improved beneficial effects over standard treatment in rat hearts after global ischemia. Scand. Cardiovasc. J. 38:245–254. 15553937 10.1080/14017430410035476

[phy213109-bib-0011] Dolinsky, V. W. , S. Chakrabarti , T. J. Pereira , T. Oka , J. Levasseur , D. Beker , et al. 2013 Resveratrol prevents hypertension and cardiac hypertrophy in hypertensive rats and mice. Biochim. Biophys. Acta 1832:1723–1733. 23707558 10.1016/j.bbadis.2013.05.018

[phy213109-bib-0012] Dolinsky, V. W. , A. Y. Chan , I. Robillard Frayne , P. E. Light , C. Des Rosiers , and J. R. Dyck . 2009 Resveratrol prevents the prohypertrophic effects of oxidative stress on LKB1. Circulation 119:1643–1652. 19289642 10.1161/CIRCULATIONAHA.108.787440

[phy213109-bib-0013] Dolinsky, V. W. , C. F. Rueda‐Clausen , J. S. Morton , S. T. Davidge , and J. R. Dyck . 2011 Continued postnatal administration of resveratrol prevents diet‐induced metabolic syndrome in rat offspring born growth restricted. Diabetes 60:2274–2284. 21810598 10.2337/db11-0374 PMC3161324

[phy213109-bib-0014] Dolinsky, V. W. , C. L. Soltys , K. J. Rogan , A. Y. Chan , J. Nagendran , S. Wang , et al. 2015 Resveratrol prevents pathological but not physiological cardiac hypertrophy. J. Mol. Med. (Berlin, Germany) 93: 413–425. 10.1007/s00109-014-1220-8 25394677

[phy213109-bib-0015] Gabriel, R. S. , and A. L. Klein . 2009 Modern evaluation of left ventricular diastolic function using Doppler echocardiography. Curr. Cardiol. Rep. 11:231–238. 19379644 10.1007/s11886-009-0033-9

[phy213109-bib-0016] Giussani, D. A. , and S. T. Davidge . 2013 Developmental programming of cardiovascular disease by prenatal hypoxia. J. Dev. Orig. Health Dis. 4:328–337. 24970726 10.1017/S204017441300010X

[phy213109-bib-0017] Goh, S. S. , O. L. Woodman , S. Pepe , A. H. Cao , C. Qin , and R. H. Ritchie . 2007 The red wine antioxidant resveratrol prevents cardiomyocyte injury following ischemia‐reperfusion via multiple sites and mechanisms. Antioxid. Redox Signal. 9:101–113. 17115889 10.1089/ars.2007.9.101

[phy213109-bib-0018] Gu, X. S. , Z. B. Wang , Z. Ye , J. P. Lei , L. Li , D. F. Su , et al. 2014 Resveratrol, an activator of SIRT1, upregulates AMPK and improves cardiac function in heart failure. Genet. Mol. Res. 13:323–335. 24535859 10.4238/2014.January.17.17

[phy213109-bib-0019] Harvey, T. J. , R. M. Murphy , J. L. Morrison , and G. S. Posterino . 2015 Maternal nutrient restriction alters Ca2 + handling properties and contractile function of isolated left ventricle bundles in male but not female juvenile rats. PLoS ONE 10:e0138388. 26406887 10.1371/journal.pone.0138388 PMC4583465

[phy213109-bib-0020] Hung, L. M. , M. J. Su , and J. K. Chen . 2004 Resveratrol protects myocardial ischemia‐reperfusion injury through both NO‐dependent and NO‐independent mechanisms. Free Radic. Biol. Med. 36:774–781. 14990356 10.1016/j.freeradbiomed.2003.12.016

[phy213109-bib-0021] Hwang, J. T. , D. Y. Kwon , O. J. Park , and M. S. Kim . 2008 Resveratrol protects ROS‐induced cell death by activating AMPK in H9c2 cardiac muscle cells. Genes Nutr. 2:323–326. 18850225 10.1007/s12263-007-0069-7 PMC2478493

[phy213109-bib-0022] Juric, D. , P. Wojciechowski , D. K. Das , and T. Netticadan . 2007 Prevention of concentric hypertrophy and diastolic impairment in aortic‐banded rats treated with resveratrol. Am. J. Physiol. Heart Circ. Physiol. 292:H2138–H2143. 17488730 10.1152/ajpheart.00852.2006

[phy213109-bib-0023] Kanamori, H. , G. Takemura , K. Goto , A. Tsujimoto , A. Ogino , T. Takeyama , et al. 2013 Resveratrol reverses remodeling in hearts with large, old myocardial infarctions through enhanced autophagy‐activating AMP kinase pathway. Am. J. Pathol. 182:701–713. 23274061 10.1016/j.ajpath.2012.11.009

[phy213109-bib-0024] Lagouge, M. , C. Argmann , Z. Gerhart‐Hines , H. Meziane , C. Lerin , F. Daussin , et al. 2006 Resveratrol improves mitochondrial function and protects against metabolic disease by activating SIRT1 and PGC‐1alpha. Cell 127:1109–1122. 17112576 10.1016/j.cell.2006.11.013

[phy213109-bib-0025] Li, Y. , Z. Cao , and H. Zhu . 2006 Upregulation of endogenous antioxidants and phase 2 enzymes by the red wine polyphenol, resveratrol in cultured aortic smooth muscle cells leads to cytoprotection against oxidative and electrophilic stress. Pharmacol. Res. 53:6–15. 16169743 10.1016/j.phrs.2005.08.002

[phy213109-bib-0026] Li, H. , N. Xia , and U. Forstermann . 2012 Cardiovascular effects and molecular targets of resveratrol. Nitric Oxide 26:102–110. 22245452 10.1016/j.niox.2011.12.006

[phy213109-bib-0027] Ojeda, N. B. , D. Grigore , L. L. Yanes , R. Iliescu , E. B. Robertson , H. Zhang , et al. 2007 Testosterone contributes to marked elevations in mean arterial pressure in adult male intrauterine growth restricted offspring. Am. J. Physiol. Regul. Integr. Comp. Physiol. 292:R758–R763. 16917022 10.1152/ajpregu.00311.2006

[phy213109-bib-0028] Raj, P. , X. L. Louis , S. J. Thandapilly , A. Movahed , S. Zieroth , and T. Netticadan . 2014 Potential of resveratrol in the treatment of heart failure. Life Sci. 95:63–71. 24361400 10.1016/j.lfs.2013.12.011

[phy213109-bib-0029] Ram, R. , D. M. Mickelsen , C. Theodoropoulos , and B. C. Blaxall . 2011 New approaches in small animal echocardiography: imaging the sounds of silence. Am. J. Physiol. Heart Circ. Physiol. 301:H1765–H1780. 21873501 10.1152/ajpheart.00559.2011 PMC3213976

[phy213109-bib-0030] Ray, P. S. , G. Maulik , G. A. Cordis , A. A. Bertelli , A. Bertelli , and D. K. Das . 1999 The red wine antioxidant resveratrol protects isolated rat hearts from ischemia reperfusion injury. Free Radic. Biol. Med. 27:160–169. 10443932 10.1016/s0891-5849(99)00063-5

[phy213109-bib-0031] Roth, G. A. , M. D. Huffman , A. E. Moran , V. Feigin , G. A. Mensah , M. Naghavi , et al. 2015 Global and regional patterns in cardiovascular mortality from 1990 to 2013. Circulation 132:1667–1678. 26503749 10.1161/CIRCULATIONAHA.114.008720

[phy213109-bib-0032] Rueda‐Clausen, C. F. , J. S. Morton , and S. T. Davidge . 2009 Effects of hypoxia‐induced intrauterine growth restriction on cardiopulmonary structure and function during adulthood. Cardiovasc. Res. 81:713–722. 19088083 10.1093/cvr/cvn341

[phy213109-bib-0033] Rueda‐Clausen, C. F. , J. S. Morton , G. D. Lopaschuk , and S. T. Davidge . 2011 Long‐term effects of intrauterine growth restriction on cardiac metabolism and susceptibility to ischaemia/reperfusion. Cardiovasc. Res. 90:285–294. 21097804 10.1093/cvr/cvq363

[phy213109-bib-0034] Rueda‐Clausen, C. F. , J. S. Morton , V. W. Dolinsky , J. R. Dyck , and S. T. Davidge . 2012 Synergistic effects of prenatal hypoxia and postnatal high‐fat diet in the development of cardiovascular pathology in young rats. Am. J. Physiol. Regul. Integr. Comp. Physiol. 303:R418–R426. 22739349 10.1152/ajpregu.00148.2012

[phy213109-bib-0035] Sahn, D. J. , A. DeMaria , J. Kisslo , and A. Weyman . 1978 Recommendations regarding quantitation in M‐mode echocardiography: results of a survey of echocardiographic measurements. Circulation 58:1072–1083. 709763 10.1161/01.cir.58.6.1072

[phy213109-bib-0036] Shah, A. , L. M. Reyes , J. S. Morton , D. Fung , J. Schneider , and S. T. Davidge . 2015 Effect of resveratrol on metabolic and cardiovascular function in male and female adult offspring exposed to prenatal hypoxia and a high‐fat diet. J. Physiol. 594:1465–1482. 26467260 10.1113/JP271133 PMC4771791

[phy213109-bib-0037] Shen, M. , R. X. Wu , L. Zhao , J. Li , H. T. Guo , R. Fan , et al. 2012 Resveratrol attenuates ischemia/reperfusion injury in neonatal cardiomyocytes and its underlying mechanism. PLoS ONE 7:e51223. 23284668 10.1371/journal.pone.0051223 PMC3527482

[phy213109-bib-0038] Sin, T. K. , A. P. Yu , B. Y. Yung , S. P. Yip , L. W. Chan , C. S. Wong , et al. 2014 Modulating effect of SIRT1 activation induced by resveratrol on Foxo1‐associated apoptotic signalling in senescent heart. J. Physiol. 592:2535–2548. 24639483 10.1113/jphysiol.2014.271387 PMC4080936

[phy213109-bib-0039] Sung, M. M. , S. K. Das , J. Levasseur , N. J. Byrne , D. Fung , T. T. Kim , et al. 2015 Resveratrol treatment of mice with pressure‐overload‐induced heart failure improves diastolic function and cardiac energy metabolism. Circ. Heart Fail. 8:128–137. 25394648 10.1161/CIRCHEARTFAILURE.114.001677

[phy213109-bib-0040] Ungvari, Z. , N. Labinskyy , P. Mukhopadhyay , J. T. Pinto , Z. Bagi , P. Ballabh , et al. 2009 Resveratrol attenuates mitochondrial oxidative stress in coronary arterial endothelial cells. Am. J. Physiol. Heart Circ. Physiol. 297:H1876–H1881. 19749157 10.1152/ajpheart.00375.2009 PMC2781360

[phy213109-bib-0041] Woods, L. L. , J. R. Ingelfinger , J. R. Nyengaard , and R. Rasch . 2001 Maternal protein restriction suppresses the newborn renin‐angiotensin system and programs adult hypertension in rats. Pediatr. Res. 49:460–467. 11264427 10.1203/00006450-200104000-00005

[phy213109-bib-0042] Xue, Q. , and L. Zhang . 2009 Prenatal hypoxia causes a sex‐dependent increase in heart susceptibility to ischemia and reperfusion injury in adult male offspring: role of protein kinase C epsilon. J. Pharmacol. Exp. Ther. 330:624–632. 19470841 10.1124/jpet.109.153239 PMC2713094

[phy213109-bib-0043] Zanetti, M. , L. V. d'Uscio , T. E. Peterson , Z. S. Katusic , and T. O'Brien . 2005 Analysis of superoxide anion production in tissue. Methods Mol. Med. 108: 65–72. 16028676 10.1385/1-59259-850-1:065

[phy213109-bib-0044] Zordoky, B. N. , I. M. Robertson , and J. R. Dyck . 2015 Preclinical and clinical evidence for the role of resveratrol in the treatment of cardiovascular diseases. Biochim. Biophys. Acta 1852:1155–1177. 25451966 10.1016/j.bbadis.2014.10.016

